# Dietary habits and physical activity patterns in relation to nutritional status among school-aged children in Pakistan: A cross-sectional study

**DOI:** 10.3934/publichealth.2023039

**Published:** 2023-06-27

**Authors:** Saleem Qureshi, Musarrat Iqbal, Azra Rafiq, Hamna Ahmed, Tooba Malik, Muhammad Nasir Kalam, Muhammad Abdullah, Qirtas Tauheed, Muhammad Daoud Butt

**Affiliations:** 1 Center for Diabetes and Liver Disease, Department of Medicine, Islamabad, Pakistan.; 2 Middle East Technical University, Ankara, Turkey.; 3 Shifa College of Medicine, Islamabad, Pakistan.; 4 Department of Public Health, Health Services Academy, Islamabad, Pakistan.; 5 Department of pharmacy, The Sahara University, Narowal, Pakistan.; 6 Department of Pharmacy, Quaid-i-Azam University, Islamabad, Pakistan.; 7 Department of pharmacy, Punjab University College of Pharmacy, Allama Iqbal Campus, University of the Punjab, 54000, Lahore.; 8 Department of Pharmacy, Faculty of Pharmacy, Bahauddin Zakariya University, 60800, Multan, Pakistan.; 9 School of Pharmaceutical Sciences, Universiti Sains Malaysia, Malaysia.

**Keywords:** nutrition, childhood malnutrition, childhood obesity, Pakistan, socioeconomic status, ow middle-income countries

## Abstract

**Background:**

Childhood malnutrition remains a significant public health problem impacting the physical and mental growth if school aged children, particularly in limited-resource countries.

**Objective:**

The study objective was to assess levels of physical activity, patterns of screen time (S.T.), the relationship between physical activity and screen time patterns, and how these factors affect growth status (adjusting for socioeconomic status).

**Methodology:**

A cross-sectional study included 3,834 children between 6–14 years attending pre-selected schools. Teachers, students, and parents were invited to fill out a standardized questionnaire, and Body Mass Index (BMI) was calculated using Center for disease control (CDC) centile charts. A Chi-square was performed to see the possible association between any height and weight abnormalities and all possible risk factors. Multivariate logistic regression was applied to see the effect of variables significantly associated with univariate analysis.

**Results:**

Approximately 2,447 (63.8%) children were between 11–14 years old and 1,387 (36.2%) were between 4–10 years old. The mean height was 143.71 ± 16.51 centimetres, the mean weight was 36.5 ± 12.9 kilogram, and the mean BMI was 17.16 ± 3.52. Multivariate logistic regression status and junk food combined affected stunting socioeconomic status was significantly associated with being underweight *p* = 0.001.

**Conclusion:**

Childhood obesity and stunting remain significant problems in Pakistani school-going children. These are significantly associated with poverty, a lack of physical activity opportunities, and available food quality.

## 1. Introduction

The twin epidemics of diabetes and obesity are still out of control globally and in Pakistan. Regarding diabetes prevalence, Pakistan was ranked 25th in 1998 and is currently 4th in the world. Children and teenagers are also experiencing an upsurge in the incidence of Type 2 diabetes mellitus [1]. An increasingly prevalent health issue is childhood obesity, which significantly contributes to this rise in incidence [2]. Additionally, recent investigations have indicated a rise in stunting among school-age children. Long-term undernutrition is measured by this linear growth retardation, which raises the chance of developing chronic illnesses as a substantial risk factor for the rise in childhood malnutrition, diabetes, obesity, stunting, socioeconomic class, and other lifestyle factors related to nutritional status [3].

Anthropometric measurements are significant indicators of nutritional status because they show growth, development, and a sufficient diet over time. Though Pakistan is a population of several different races, there is little information on various growth patterns and deficiencies [4]. While Saudi Arabia, Turkey, and Iran have their respective development charts, most rely on either the WHO Regional Office Eastern Mediterranean (EMRO) child and adolescent health charts or the Center for Disease Control (CDC) centile charts [5]. Centile charts have recently been created in Pakistan but must be validated and accepted. It is necessary to determine how lifestyle and demographic factors affect the growth traits of school-age children. Only then would it be possible to implement national initiatives to stop the rising incidence of these developmental and growth defects and stop the diabetes and obesity epidemics at an early stage. The effectiveness of preventative interventions, such as lifestyle changes emphasizing healthy eating, weight loss, and increased physical activity, is well supported by data. Results from National Diabetes Prevention Programs (DPP) worldwide, including those in the US, Finland, China, and India, have improved significantly [6].

By lowering individual productivity, stunting has enormous and varied implications for a county's economy [7]. For instance, the study found that many economists attempt to stifle growth, which can reduce a nation's GDP by up to 3%. As a developing nation, Pakistan has a sizable number of cases of child stunting; this study carefully upheld SDG Goal 3 and addressed the problem of stunted growth related to a health challenge [8]. This research will assist policymakers in developing future guidelines for preventing stunting growth as Pakistan prioritizes making numerous attempts to achieve the SDGs. Stunting growth is a significant obstacle to achieving SDG 3 (Good health and well-being), which is a target in the 2030 Agenda for Sustainable Development [7].

However, the paper's commitment is to conduct a comprehensive study on the issue and promises to implement urgent efforts to address the growth stunting among children affected by socioeconomic demographic (SED) characteristics. Pakistan Demographic and Health Survey (PDHS) is the primary data source for the government on health in Pakistan, and despite organizational commitment, efforts without evidence would be fruitless. The policy gap covered by this study used this survey for a systematic analysis to offer evidence-based policy recommendations and build initiatives. This study examines stunting in children under five concerning SED variables. After considering several factors and variables from the PDHS and additional data, it was decided that the food and health departments may implement a brief guideline to prevent stunting growth in children. For instance, according to Zaidi et al. (2013), united nation organization (UNO) advised the local government to control stunting growth by implementing sound health policies and SED variables [9]. The study aims to assess levels of physical activity, patterns of screen time (S.T.), the relationship between physical activity and screen time patterns, and how these factors affect growth status (adjusting for socioeconomic status).

## 2. Methodology

### 2.1. Study site

A cross-sectional descriptive study was conducted in 22 pre-selected schools in the Punjab Province of Pakistan. The chosen schools provided written informed permission, and the head teacher received information about the study's context. The study covered public and private schools from urban and rural locations from February 2021 through July 2022.

### 2.2. Sample size

The study was comprised of 3834 students ranging from pre-school to high school.

The sample size is calculated using the formula:

n=Nx/[(N−1)E2+x)] (1)

where n=sample size, N=Population size, x=Factor of calculation and E=margin of error.

Using the Raosoft formula suggests a minimum sample size of n= 161 from each selected school. A convenient sampling approach was used to have a minimum number of the inclusion criteria, include those who were of aged 4 to 14 years, either male or female, have the ability to communicate correctly, do not have any metabolic syndrome associated, they must be regular students of a particular school, and their parents were present at the last 2 parent-teachers' meetings. Students who were excluded from the data collection included those who suffered from any accident in the past 2 years that affected their overall health state, those having less than 6 months of tenure in the selected school, and those suffering from a genetic disease state affecting their overall growth.

### 2.3. Invitation and data collection

After the initial screening, students were given invitation letters for their parents to attend the parent-teacher meeting on the dates mentioned. With the assistance of their class teachers, the students and parents completed a validated structured questionnaire to evaluate their socioeconomic position, dietary preferences, amount of physical activity, and screen time. A full-day workshop was organized to give all respective class teachers hands-on, basic training for the questionnaires to be filled. Qualified technicians made anthropometric measurements in the classroom following standard guidelines. The assigned field team in the group was tasked to collect and validate the information gathered during the activity; the field team was comprised of A.R., H.A., TM, MNK, MA, and Q.T.

Parents and legal guardians of every child enrolled in the study were interviewed to obtain the sociodemographic information, which was then entered into a pre-structured questionnaire. The questionnaire was validated using 20% of the sample, revised accordingly, and after final validation from the student sample, teachers, and physicians. The questionnaire was translated into Urdu (Appendix A) using the standard forward-backwards process, yielding a Cronbach alpha of 0.631, within the appropriate range (0.45–0.9).

The data collected comprised of the child's biographical details, nutritional background, and family/social background. Using the methodology suggested by Olusanya et al., each kid was allocated a socioeconomic class based on the father's profession and the mother's level of education: social classes I and II represented the upper socioeconomic class; social class III represented the middle socioeconomic class; and social classes IV and V represented the lower socioeconomic class [10].

### 2.4. Ethical approval and consent to participate

The Shaheed Zulfkar Ali Bhutto Medical University's Ethical Review Board (3–17/2015/ERB/SZABMU) approved the study. All subjects who were a part of this study provided their informed permission. This study was conducted in accordance with the Declaration of Helsinki. The standards for “The Strengthening the Reporting of Observational Studies in Epidemiology (STROBE)” were followed when reporting the current study. Attached is Appendix B.

### 2.5. Analysis

The statistical software SPSS®, version 20.0.0, was used to clean, code, and analyze the data (IBM Corp, Armonk, NY, USA). The WHO Anthro software, version 2, (http://www.who.int/childgrowth/software/en/) was used to enter and evaluate the gathered anthropometric data. Averages and standard deviation were calculated for continuous variables, whereas ratios and proportions were computed for categorical variables. When appropriate, Fisher's exact or Pearson's chi-square test was used to examine the relationship between categorical variables. The chance of a child experiencing a developmental delay due to malnutrition was calculated using odds ratios with 95% confidence intervals. The developmental delay was evaluated using Denver Developmental Screening Test (DDST). In comparing studies, a *p* value of less than 0.05 was considered statistically significant. A multivariable logistic regression was used to examine the effects of factors significantly related in univariate analysis and was provided as O.R.s with 95% C.I.s. To handle the issue of zero counts in some cells for categorical variables, we used a statistical method that accounted for this issue. Specifically, we used an exact logistic regression, which calculates the exact probability of observing the data given the null hypothesis of no association between the predictor and the outcome variable.

## 3. Results

Of the 3834 students, 2624 (68.4%) were males and 1210 (31.6%) were females. The mean age was 11.30 ± 2.72 years. Approximately 2447 (63.8%) children were between 11–14 years old and 1387 (36.2%) were between 4–10 years old. The mean height was 143.71 ± 16.51 cms, the mean weight was 36.50 ± 12.9 kgs, and the mean BMI was 17.16 ± 3.52. The number of children in the low, middle, and high socioeconomic classes were 1428 (38.7%), 1516 (39.5%), and 836 (21.8%), respectively. (Table 1). Approximately 2459 (64.1%) children had 3 to 4 meals daily, while 1375 (39.5%) reported having two meals/per day.

All the participants reported a daily intake of junk food. Of these, 2366 (61.7%) eat junk food more than twice a day and 1468 (38.3%) eat junk food once a day. In the study population, 1217 (31.7%) children reported a lack of physical activity and 1369 (35.7%) reported ≤1 hour of physical activity per day. Only 1248 (32.6%) spent more significant than 1 hour in physical sports and games and 1217 (31.7%) were having no physical activity at all. More than 1 hour of screen time was seen for 1504 (39.2) students. The year-wise height for age and weight for age are given in Table 2.

A chi-square test was performed to see the association between height, weight, BMI, and lifestyle variables/risk factors. The overall prevalence of stunting was higher in children within the low socioeconomic group, with 169 (11.4%) of students, compared to those in the middle and high socioeconomic groups, with 19 (1.3%) and 4 (0.5%) of students, respectively. Additionally, the Z score for height and age were similarly significantly related to the low socioeconomic class (*p* = 0.001), see Table 3.

Overall stunting based on height for age was present in 192 (5%) of students within the total cohort. Of these, 106 (55.2%) were male and 86 (44.8%) were female. Approximately 189 (98.4 %) of stunted children were 4–10 years old and 3 (1.6%) students were 10-14 years old. A total of 165 (4.3%) children were overweight; out of these, 163 (6.7%) were in the 11–14 years group. The number of underweight children was 8 (0.2%). The Z scores for height were -0.83 ± 0.77 (Mean ± S.D.) and -0.97 ± 0.91 in males and females, respectively, in the 4–10 years age group, and were 0.52 ± 0.74 and 0.44 ± 0.61 in males and females, respectively, in the 11–14 years group (Table 4). The Z scores for weight were -0.74 ± 0.59 and -0.76 ± 0.73 in males & females, respectively, in the 4–10 years age group, and were 0.41 ± 0.92 and 0.46 ± 0.91 in males and females, respectively, in the 11–14 years group. The Z scores for BMI were -0.42 ± 0.77 and -0.37 ± 0.86 in males and females, respectively, in the 4–10 years age group, and were 0.18 ± 1.00 and 0.35 ± 1.09 in males and females, respectively, in the 11–14 years age group (Table 4).

**Table 1.** Background and demographic characteristics of participants.

Attribute		Descriptive
Gender [n (%)]	Male	2624 (68.4)
	Female	1210 (31.6)
Age (mean ± S.D)	11.30 ± 2.72
Age years [n (%)]	4–10	1387 (36.2)
	10.1–14	2447 (63.8)
Height (mean ± S.D)	143.71±16.51
Weight (mean ± S.D)	36.50 ± 12.9
BMI (mean ± S.D)	17.16 ± 3.52
Socioeconomic status [n (%)]	High income	836 (21.8)
	Middle income	1516 (39.5)
	Low income	1482 (38.7)
Frequency of meal intake [n (%)]	≤ 2	1375 (35.9)
	>2	2459 (64.1)
Frequency of junk intake [n (%)]	≤ 1	1468 (38.3)
	> 1	2366 (61.7)
Hours of physical activity [n (%)]	Nil	1217 (31.7)
	≤ 1 hour	1369 (35.7)
	>1 hour	1248 (32.6)
Use of screen time [n (%)]	≤ 1 hour	2330 (60.8)
	>1 hour	1504 (39.2)
Height for age [n (%)]	Stunted	192 (5.0)
	Normal	3592 (93.7)
	Tall	50 (1.3)
Weight for age [n (%)]	Underweight	8 (0.2)
	Normal	3661 (95.5)
	Overweight	165 (4.3)
BMI for age Z score [n (%)]	Underweight	18 (0.5)
	Normal	3535 (92.2)
	Overweight	281 (7.3)

Out of all the underweight children, 8 (100%) children belonged to the low socioeconomic class. Interestingly, the incidence of overweight children was also higher in this socioeconomic strata with a *p* < 0.001. Age, frequency of meal intake, and hours of physical activity all showed a strong association with stunting and obesity. Gender was significantly associated with stunting (*p* < 0.001) but not obesity (*p* = 0.31). The frequency of junk food intake and screen time use was significantly associated with stunting (*p* < 0.001) but not weight abnormalities, as seen in Table 5.

**Table 2.** Anthropometric data according to age.

Age Years	Gender					
	Anthropometric data of males			Anthropometric data of females		
	Weight	Height	BMI	Weight	Height	BMI
4.0	14.8 ± 3.9	100.2 ± 7.7	14.55 ± 2.14	12.9 ± 2.0	97.6 ± 6.6	13.54 ± 1.45
5.0	16.8 ± 2.3	107.1 ± 7.1	14.66 ± 1.95	15.5 ± 2.6	106.2 ± 6.9	13.69 ± 1.83
6.0	19.0 ± 3.4	116.0 ± 10.5	13.85 ± 3.15	18.6 ± 3.9	113.3 ± 6.4	14.37 ± 1.99
7.0	22.5 ± 5.0	123.5 ± 11.2	14.78 ± 2.63	21.9 ± 5.8	118.6 ± 8.7	15.37 ± 2.60
8.0	26.0 ± 6.1	128.1 ± 8.1	15.68 ± 2.40	26.0 ± 5.6	128.6 ± 8.8	15.66 ± 2.63
9.0	28.3 ± 6.2	133.3 ± 7.9	15.83 ± 2.56	31.1 ± 7.6	136.2 ± 8.9	16.62 ± 3.09
10.0	30.6 ± 7.2	137.5 ± 7.7	16.11 ± 3.05	32.5 ± 9.4	137.9 ± 9.2	16.83 ± 3.38
11.0	35.0 ± 9.3	143.6 ± 11.1	16.83 ± 3.26	34.6 ± 8.4	141.2 ± 8.2	17.19 ± 3.15
12.0	38.7 ± 10.1	148.4 ± 10.0	17.44 ± 3.37	37.9 ± 10.6	146.1 ± 8.7	17.52 ± 3.78
13.0	42.7 ± 11.1	153.3 ± 10.1	17.95 ± 3.64	41.8 ± 9.7	152.0 ± 7.4	17.99 ± 3.35
14.0	46.7 ± 11.8	158.5 ± 9.0	18.44 ± 3.66	46.5 ± 11.4	155.3 ± 6.4	19.18 ± 4.12

**Table 3.** Association between different anthropometrical attributes and sociodemographic strata.

Attribute	All frequencies [n (%)]	High socioeconomic [n (%)]	Middle frequency [n (%)]	Low frequency [n (%)]	*p* value
Height for age Z-score
Stunted	192 (5.0)	4 (0.5)	19 (1.3)	169 (11.4)	0.001*
Normal	3592 (93.7)	830 (99.3)	1474 (97.2)	1288 (86.9)	
Tall	50 (1.3)	2 (0.2)	23 (1.5)	25 (1.7)	
BMI for age Z-score
Underweight	18 (0.5)	0 (0.0)	3 (0.2)	15 (1.0)	0.001*
Normal	3535 (92.2)	799 (95.6)	1373 (90.6)	1363 (92.0)	
Obese	281 (7.3)	37 (4.4)	140 (9.2)	104 (7.0)	

Note: Pearson's Chi-square test was performed to evaluate the association among categories. *p < 0.05 is considered as significant.

**Table 4.** Z score in school children 4–14 years of age for height, weight, and BMI.

Project	Age group			
	4–10		10–14	
	Male	Female	Male	Female
Height	-0.83 ± 0.77	-0.97 ± 0.91	0.52 ± 0.74	0.44 ± 0.61
Weight	-0.74 ± 0.59	-0.76 ± 0.73	0.41 ± 0.92	0.46 ± 0.91
BMI	-0.42 ± 0.77	-0.37 ± 0.86	0.18 ± 1.00	0.35 ± 1.09

A multivariate logistic regression was applied to see the strength of the association of variables that had a significant association in the univariate analysis. Socioeconomic status and junk food combined affected stunting *OR* 8.141 (5.56–11.9), whereas socioeconomic status was significantly associated with underweight *OR* 5.961(2.048–17.351), *p* = 0.001, see Table 6.

## 4. Discussion

The study included 3834 students: 68.4% males and 31.6% females. The mean age was 11.3 ± 2.72 years, with 63.8% of children in the 11–14 age range and 36.2% in the 4–10 age range. The mean height was 143.71 ± 16.51 cms, the mean weight was 36.5 ± 12.9 kgs, and the mean BMI was 17.16 ± 3.52. Among the participants, 38.7%, 39.5%, and 21.8% belonged to low, middle, and high socioeconomic classes, respectively. Most children reported having 3–4 meals daily, and all reported a daily intake of junk food, with 61.7% consuming it more than twice a day. The prevalence of stunting was 5%, with 11.4% of affected children in the low socioeconomic class. The prevalence of overweight was 4.3%, with most cases (6.7%) in the 11–14 age group. A multivariate logistic regression analysis showed a strong association between stunting, low socioeconomic status, and junk food intake (*OR* 8.141, 95% *CI* 5.56–11.9). Overall, the findings highlight the high prevalence of unhealthy lifestyle habits among school children in this population and the urgent need for effective interventions to address this issue.

Worldwide, there is a severe public health concern over childhood obesity. Obesity among school-age children and adolescents has increased from 11 million to 124 million people (2016 estimates). In 2016, it was also predicted that 216 million people were overweight but not obese [10]. The condition also affects younger children; in 2017, almost 38 million of children under 5 years old were overweight or obese [11]. Approximately, 42 million Southeast Asian children aged 5 to 9 were overweight or obese in 2016, according to data conducted by the WHO. The prevalence increased by 21% between 2010 and 2016 [12].

A third of all children under five are stunted in developing or emerging countries, where child malnutrition has decreased overall but still poses a severe public health concern; 70% of these children reside in South Asia [13]. Pakistan's stunting rates dropped from 47% in 1980 to 33% in 2000. Stunting is a severe concern in Pakistan, affecting 38% of children under five, one of the highest rates in the world [14]. In Sindh, 50% of kids exhibit signs of stunting. In our sample, children aged 4 to 10 constituted most of those with overall stunting (13.6%), and girls were more affected than males (7.1% vs 4%). According to Sina Aziz et al. [15], 14% of Pakistan's pediatric population is stunted. According to the Z score (>2SD above -1.96), the prevalence of obese children in our study was 4.3% (n = 165). The 5% observed study that was carried out in Pakistan is comparable [16]. According to Kwabla et al., stunting in children can reach as high as 67% and is more prevalent in boys than girls between the ages of 4 and 10, with a *p* value of 0.001. Approximately 15% of the population (n = 580) had a BMI that was found to be either overweight (85th–95th centile) or obese (>95th centile). Some have asserted that up to 14% of people are overtly obese. This prevalence is higher than the 0.8% reported in other studies [17].

The primary outcome variables of overweight/obesity and stunting were assessed, together with possible risk factors (socioeconomic status, age, frequency of meal intake, and hours of physical activity). Only 67.4% of young people in our poll said they engaged in less than an hour of physical activity every day. Only 32.6% of kids played games daily for over an hour. In southern Pakistan, 30% of kids reported engaging in physical activity, such as field games, jogging, and cycling, while only 5% of obese kids actually performed. Throughout time, socioeconomic anomalies impact childhood stunting and weight status. In addition to income, it also considers how many meals are eaten each day, the type of flooring used, such as mud as opposed to cement, brick, or wood, and the amount of meat and dairy products consumed.

According to earlier research, age, socioeconomic status, how often people eat meals, and the number of hours they exercise all showed a significant link (*p* = 0.001 for each item). In addition, there was no correlation between height, weight, gender, the frequency of eating junk food (*p* = 0.16), and the amount of time spent on screens per hour (*p* = 0.78) [18].

Compared to children in the medium and high socioeconomic categories, approximately19 (9.9%) and 4 (2.1%), respectively, children in the low socioeconomic group (169, 88%) had a greater overall prevalence of stunting. The socioeconomic class was significantly linked with the Z scores for height and age (*p* = 0.001) [19].

According to recent research, stunting and underweight among school children aged 5 to 10 from urban squatter settlements in Islamabad were both common, with a frequency of 29.5% and 35%, respectively. In our study, 72.1% (n = 119) of the boys were obese and 55.2% (n = 106) were stunted. According to Riaz et al., female stunting and underweight rates were 29.1% and 8.5%, respectively. None of the women were overweight. Underweight, stunting, and obesity were each present in 19.6%, 12.8%, and 0.6% of boys, respectively [20]. In a recent study of Nepali schoolchildren in Kathmandu, stunting and underweight were found in 43.6% of female pupils and 1.8% of male students [21].

Sedentary behaviors have been linked to an increased risk of cardio-metabolic disease, all-cause mortality, and various physiological and psychological problems, regardless of physical activity levels. Hence, to optimize health benefits, initiatives to address the issue of inactivity should increase planned physical activity and reduce sedentary behaviors, particularly in the pediatric population [22].

Teenagers' levels of physical exercise are insufficient, with 81% of adolescents worldwide (78% males, 84% females) not meeting the minimum guidelines. Low levels of physical activity were observed in low-middle- and high-income countries as well as WHO regions [23]. Obesity in children is correlated with physical inactivity. Nonetheless, levels of physical activity are still low across all age groups. Current research shows that just 7% of children aged 6 to 19 engage in up to 60 minutes of moderate-vigorous daily exercise [24].

In our study, 67.4% of the children engaged in physical activity less than an hour daily. Only 32.6% said they engaged in physical activities, including field games, running, and cycling for over an hour daily. Only 29.7% of obese kids exercised for more than an hour, and only 70.3% of obese kids reported engaging in physical activity. Only 5% of obese kids actually engaged in physical activity [25].

With earlier studies showing a higher prevalence of obesity with higher socioeconomic levels, this finding is neither consistent nor compatible with that work. Additionally, parents' perceptions of their children's nutritional status, such as being underweight, overweight, or obese, may be inaccurate [26]. This explains why informing parents about nutrition and the proper height and weight for kids is essential. Due to the lack of randomization in the schools chosen for the study, the findings of our study and those of other studies cannot be generalized. Additionally, the study design must consider the large number of kids who do not attend school.

**Table 5.** Factors Associated with height and weight categories of school-age children.

Attributes	Stunting [n (%)]	Normal [n (%)]	Over growths [n (%)]	*p* value	Underweight [n (%)]	Normal [n (%)]	Obese [n (%)]	*p* value
**Socioeconomic**								
High	4 (2.1)	830 (23.1)	2 (4.0)	0.042*	0 (0.0)	822 (22.5)	14 (8.5)	0.039*
Middle	19 (9.9)	1474 (41.0)	23 (46.0)		0 (0.0)	1444 (39.4)	72 (43.6)	
Low	169 (88.0)	1288 (35.9)	25 (50.0)		8 (100.0)	1395 (38.1)	79 (47.9)	
**Gender**								
Male	106 (55.2)	2480 (69.0)	38 (76.0)	0.011*	4 (50.0)	2501 (68.3)	119 (72.1)	0.071
Female	86 (44.8)	1112 (31.0)	12 (24.0)		4 (50.0)	1160 (31.7)	46 (27.9)	
**Age**								
4–10	189 (98.4)	1198 (33.4)	0 (0.0)	0.001*	8 (100.0)	1377 (37.6)	2 (1.2)	0.001*
10–14	3 (1.6)	2394 (66.6)	50 (100.0)		0 (0.0)	2284 (62.4)	163 (98.8)	
**Frequency of meal intake/day**
<2	70 (36.5)	1274 (35.5)	31 (62.0)	0.021*	5 (62.5)	1296 (35.4)	74 (44.8)	0.029*
≥2	122 (63.5)	2318 (64.5)	19 (38.0)		3 (37.5)	2365 (64.6)	91 (55.2)	
**Frequency of junk intake/day**
<1	39 (20.3)	1399 (38.9)	30 (60.0)	0.001*	2 (25.0)	1392 (38.0)	74 (44.8)	0.33
≥1	153 (79.7)	2193 (61.1)	20 (40.0)		6 (75.0)	2269 (62.0)	91 (55.2)	
**Hours of physical activity/day**
Nil	43 (22.4)	1150 (32.0)	24 (48.0)	0.0001*	3 (37.5)	1147 (31.3)	67 (40.6)	0.021*
<1	117 (60.9)	1245 (34.7)	7 (14.0)		5 (62.5)	1315 (35.9)	49 (29.7)	
≥1	32 (16.7)	1197 (33.3)	19 (38.0)		0 (0.0)	1199 (32.8)	49 (29.7)	
**Use of screen time /day**
<1 hour	164 (85.4)	2132 (59.4)	34 (68.0)	0.052	5 (62.5)	2229 (60.9)	96 (58.2)	0.66
≥1 hour	28 (14.6)	1460 (40.6)	16 (32.0)		3 (37.5)	1432 (39.1)	69 (41.8)	

Note: (1) Fisher Exact test was applied, *significance defined as *p* < 0.05. (2) Stunting is defined as height-for-age Z-score (HAZ) < -2; underweight is defined as weight for height Z-score (WHZ) < −2, and Overweight/Obesity is defined as BMI-for-Age > +1SD.

**Table 6.** Multivariate regression analysis keeping height and weight as the dependent variable.

Project		*p* value	Odds Ratio	95% confidence interval for *OR*	
				Lower Bound	Upper Bound
Stunted	Socio-economic status	<0.001	8.141	5.556	11.929
	Age group	<0.001	0.006	0.002	0.019
	Meal/day	0.044	0.653	0.431	0.988
	Hours of physical activity	0.076	0.766	0.571	1.028
	Screen hours	<0.001	0.371	0.234	0.590
	Junk food frequency	<0.001	2.506	1.535	4.094
Underweight	Socio-economic status	0.001	5.961	2.048	17.351
	Age group	0.007	0.244	0.087	0.680
	Meal/day	0.823	0.877	0.277	2.776
	Hours of physical activity	0.348	1.432	0.677	3.027
	Screen hours	0.383	1.557	0.575	4.217
	Junk food frequency	0.091	6.002	0.749	48.092

Children who ate no more than three meals per day had a higher prevalence of risk factors for underweight, stunting, and wasting compared to children who ate more than three meals daily. According to a study conducted in Kenya, children who lived in homes with mud floors were likelier to stunt than those with cement, brick, ceramic, or wood floors. The mud floor refers to the parents' low social standing [27].

There is overwhelming evidence that malnutrition and the associated morbidity and mortality often affect children in lower socioeconomic groups, particularly in developing nations. Males had a higher chance of being underweight by about 1.48 (95% *CI*: 1.01–2.30, *p* = 0.05) compared to girls. This somewhat contradicts the widely held belief that girls in Southeast Asia are more likely than boys to be underweight [28].

The table shows that males' risk of stunting was approximately 1.64 (1.09–2.23) times higher than girls' [29]. Stunting is likely more common in boys than girls, just as being underweight. The prevalence of stunting is similar to that of underweight economic conditions, as it was shown that children in the low and middle-income groups had a higher risk of stunting than those in the high-income group [30]. The risk of stunting was approximately 2.64 (95% *CI*: 1.52–4.56, *p* = 0.001) times higher for children in the low-income group than those in the high-income group. Children in the middle-income group also had around 2.18 (95% *CI*: 1.13–4.19, *p* = 0.022) times higher odds of stunting than those in the high-income group [31].

In Pakistan, 63.3% of the underweight children were in the lower SEC compared to 70% of the obese children who belonged to the higher SEC. This result aligns with previous research showing that socioeconomic class impacts obesity rates in undeveloped countries [32]–[34]. Similarly, 51.8% of children were overweight. This is comparable to earlier research, which found that underweight rates in boys aged 2–5 and 6–10 were 26 (21%) and 30 (90%), respectively [35]. For girls, these frequencies are 21.74% and 24.37%, respectively. Compared to children in the high-income group, those in the low-income group had about 2.58 (95% *CI*: 1.32–5.05, *p* = 0.006) times higher likelihood of being underweight. Children in the middle-income group showed about 2.41 (95% *CI*: 1.41–5.24, *p* = 0.027) similar results [36].

## 5. Conclusion

In conclusion, our study highlights the significant impact of socioeconomic determinants on childhood obesity in Pakistan. Our findings indicate that obesity rates are higher among children from higher SES households, while underweight rates are higher among children from lower SES households. This suggests that addressing inequalities in income and education may be critical in reducing childhood obesity in the country.

Comparing the situation in Pakistan to developed nations, we can observe similar patterns where childhood obesity rates are higher among children from higher SES households. This highlights the need for a global approach to addressing childhood obesity and improving nutrition status.

Overall, our study underscores the importance of public health initiatives that focus on promoting healthy dietary choices and physical activity. In addition, future research should explore the effectiveness of integrated strategies that target both underweight and overweight children, particularly in developing nations like Pakistan. By educating youngsters about the importance of healthy lifestyles and addressing socioeconomic disparities, we can work towards reducing the dual burden of disease and promoting better health outcomes for children.

## 6. Future Recommendations

The findings of this research have important implications for the psychology and mental health field. The results suggest that early childhood experiences may significantly impact an individual's mental health and well-being in adulthood. Future research could explore how childhood experiences influence adult mental health outcomes and identify potential interventions to prevent or mitigate adverse effects. Additionally, these findings may have practical implications for mental health practitioners, as they highlight the importance of assessing and addressing early life experiences when treating individuals with mental health concerns. Overall, this research underscores the importance of understanding the long-term effects of early childhood experiences on adult mental health and well-being.

## 7. Limitations

The absence of randomization in school selection constrained the study's generalizability. However, the schools that gave their assent demonstrated a wide range in the student's socioeconomic status, representing Pakistani society. Children who do not have access to education were not taken into account in the study design. Undernutrition and obesity coexist among schoolchildren in Pakistan.
