# Supplementary material for: Dietary habits and physical activity patterns in relation to nutritional status among school-aged children in Pakistan: A cross-sectional study
Source: AIMS Public Health. 2023 Jun 27;10(3):553–67. doi: 10.3934/publichealth.2023039 (PMC10567976; doi:10.3934/publichealth.2023039)
Supplement: Supplementary file 1 [file publichealth-10-03-039-s001.pdf]

Public Health

AIMS Public Health, 10(3): 553–567.
DOI: 10.3934/publichealth.2023039
Received: 19 March 2023
Revised: 06 June 2023
Accepted: 07 June 2023
Published: 27 June 2023

http://www.aimspress.com/journal/aimsph

Research article

Dietary habits and physical activity patterns in relation to nutritional status among school-aged children in Pakistan: A cross-sectional study

Saleem Qureshi[1], Musarrat Iqbal[1], Azra Rafiq[2],Hamna Ahmed[3], Tooba Malik[4], Muhammad Nasir Kalam[5,6], Muhammad Abdullah[7], Qirtas Tauheed[8] and Muhammad Daoud Butt[6,9,]*

[1] Center for Diabetes and Liver Disease, Department of Medicine, Islamabad, Pakistan.
[2] Middle East Technical University, Ankara, Turkey.
[3] Shifa College of Medicine, Islamabad, Pakistan.
[4] Department of Public Health, Health Services Academy, Islamabad, Pakistan.
[5] Department of pharmacy, The Sahara University, Narowal, Pakistan.
[6] Department of Pharmacy, Quaid-i-Azam University, Islamabad, Pakistan.
[7] Department of pharmacy, Punjab University College of Pharmacy, Allama Iqbal Campus, University of the Punjab, 54000, Lahore.
[8] Department of Pharmacy, Faculty of Pharmacy, Bahauddin Zakariya University, 60800, Multan, Pakistan.
[9] School of Pharmaceutical Sciences, Universiti Sains Malaysia, Malaysia.

* **Correspondence:** Email: Muhammaddaoud10@yahoo.com, daoudbutt@student.usm.my.

Supplementary I. Nutritional Awareness Camp – Student Questionnaire.

All this information will be kept **Confidential**.	Date: __________________
Student Information	
Name:	Son/Daughter of:

Continued on next page

Date of birth: Gender: Male ☐ Female ☐

Socioeconomic Class: I ☐ II ☐ III ☐ IV ☐ V ☐

Education:	Class:	Section:
Height:	Weight:	BMI:
Waist Circumference:	Hip Circumference:	WHR:

Daily Activity	
How many meals per day?	1 ☐ 2 ☐ 3 ☐
Do you eat snacks while watching TV?	Yes ☐ No ☐
How many snacks per day?	0 ☐ 1 ☐ 2 ☐ 3 ☐ 4 ☐
How many servings of grains (paratha, roti, naan, bread, rice, dalia) per day?	1☐ 2 ☐ 3 ☐
How many servings (3oz) of chicken /mutton /beaf per day?	1☐ 2 ☐ 3 ☐
How many servings (1/2 cup) of vegetables per day?	1☐ 2 ☐ 3 ☐
How many fruits per day?	1☐ 2 ☐ 3 ☐
How many glasses (8 oz) of water per day?	1☐ 2 ☐ 3 ☐ 4☐ 5 ☐ ≤5 ☐
What type of water do you drink?	Tap ☐ Well☐ Filtered☐ Boiled ☐ Mineral ☐
How many glasses (8 oz) of milk per day?	1☐ 2 ☐ 3 ☐
How many juices per day?	0 ☐ 1 ☐ 2 ☐ 3 ☐ 4 ☐
How many hours of physical activity per day?	0 ☐ 1–2Hrs ☐ 3–4Hrs ☐ <5Hrs ☐
What type of physical activity per day?	Walking ☐ Running ☐ Cycling ☐ Swimming ☐ Football ☐ Cricket ☐ Others_______________
How many screen hours (television, laptop, mobiles) per day?	0–1Hrs ☐ 2–3Hrs ☐ 3–4Hrs ☐ <4Hrs ☐

Weekly Activity:

1. How many servings (3oz) of red meat per week?

___

2. How many servings (3oz) of chicken per week?

___

3. How many servings (3oz) of fish per week?

___

4. How many desserts or sweets per week?

___

5. How many bottles (500 ml) of cold drinks per week?

___

6. How many meals are eaten away from home per week?

___

7. What are the types of restaurants you visit?

___

Supplement II. STROBE Statement—Checklist of items that should be included in reports of cross-sectional studies.

Project	Item No	Recommendation	Page No.	Relevant text from manuscript
**Title and abstract**	1	(*a*) Indicate the study's design with a commonly used term in the title or the abstract	1	a cross-sectional study
		(*b*) Provide in the abstract an informative and balanced summary of what was done and what was found	2–3	Childhood obesity and stunting remain significant issues among Pakistani school children, with poverty, limited physical activity opportunities, and low-quality food availability playing contributing roles.
**Introduction**				
Background/rationale	2	Explain the scientific background and rationale for the investigation being reported	2–4	Childhood malnutrition remains a significant public health problem impacting school-aged children's physical and mental growth, particularly in limited-resource countries.
Objectives	3	State specific objectives, including any prespecified hypotheses	4	To evaluate the factor associated with obesity and stunting in Pakistani school children (4–14 years) and to assess the effect of socioeconomic status, dietary habits, and exercise patterns on the outcome by evaluating anthropometric data.
**Methods**				
Study design	4	Present key elements of study design early in the paper	4–6	This was a cross-sectional observational study

Continued on next page

Setting	5	Describe the setting, locations, and relevant dates, including periods of recruitment, exposure, follow-up, and data collection	4–5	A cross-sectional descriptive study was conducted in 22 pre-selected schools in the Punjab Province of Pakistan. The chosen schools provided written informed permission, and the head teacher received information about the study's context. The study covered public and private schools from urban and rural locations
Participants	6	(*a*) Give the eligibility criteria, and the sources and methods of selection of participants	4–6	include those who were of age 4 to 14 years regardless of gender, either male or female, the children able to communicate correctly, do not have any metabolic syndrome associated, the student must be regular students at the particular school, and their parents were present at the last 2 parent teachers' meetings.
Variables	7	Clearly define all outcomes, exposures, predictors, potential confounders, and effect modifiers. Give diagnostic criteria, if applicable	6	The primary aim of the study was to assess children's overall growth in relevance with their Age, Gender, Physical activity, Screen time, Socioeconomic status, BMI, Weight, and Height.
Data sources/ measurement	8*	For each variable of interest, give sources of data and details of assessment methods (measurement). Describe comparability of assessment methods if there is more than one group	*6*	The data was collected from the children in their routine school activities. With the assistance of their class teachers, the students and parents completed a validated structured questionnaire to evaluate their socioeconomic position, dietary preferences, amount of physical activity, and screen time.
Bias	9	Describe any efforts to address potential sources of bias	6	To address the potential bias, the data collection process was completely done by the third individual not involved in the study. However, to keep data integrity, the investigator team was aligned with the group to check for data validity.
Study size	10	Explain how the study size was arrived at	6	Using the Raosoft formula suggests a minimum sample size of n= 161 from each selected school. The study comprised 3834 students in grades pre-school to high school.

Continued on next page

Quantitative variables	11	Explain how quantitative variables were handled in the analyses. If applicable, describe which groupings were chosen and why	6	Age, BMI, Weight, and Height were the quantitative variables selected for the study.
Statistical methods	12	(*a*) Describe all statistical methods, including those used to control for confounding	6	Averages and standard deviation were calculated for continuous variables, whereas ratios and proportions were computed for categorical variables. When appropriate, Fisher's exact or Pearson's chi-square test was used to examine the relationship between categorical variables.
		(*b*) Describe any methods used to examine subgroups and interactions	6	Multivariable logistic regression was used to examine the effects of factors significantly related in univariate analysis and was provided as O.R.s with 95% C.I.s.
		(*c*) Explain how missing data were addressed	Not Applicable	
		(*d*) If applicable, describe analytical methods taking account of sampling strategy	Not Applicable	
		(*e*) Describe any sensitivity analyses	Not Applicable	
**Results**				
Participants	13*	(a) Report numbers of individuals at each stage of study—eg numbers potentially eligible, examined for eligibility, confirmed eligible, included in the study, completing follow-up, and analysed	7–10	Of 3834 students, 2624(68.4%) were males, and 1210 (31.6%) were females.
		(b) Give reasons for non-participation at each stage	Not Applicable	Not Applicable
		(c) Consider use of a flow diagram	Not Applicable	Not Applciable

Continued on next page

Descriptive data	14*	(a) Give characteristics of study participants (eg demographic, clinical, social) and information on exposures and potential confounders	7–8	The mean age was 11.3 ± 2.72 years. The majority of children, 2447 (63.8%), were 10–14 years, and 1387 (36.2%) were 4–10 years old. The mean height was 143.71± 16.51 cms, the mean weight was 36.5 ± 12.9 kgs, and the mean BMI was 17.16±3.52. The number of children in the low, middle & high socioeconomic classes was 1428 (38.7%), 1516 (39.5%), and 836 (21.8%), respectively. (Table 1). Most of the children, 2459 (64.1%), had 3 to 4 meals daily, while 1375 (39.5%) reported having two meals/per day.
		(b) Indicate number of participants with missing data for each variable of interest		Not Applicable
Outcome data	15*	Report numbers of outcome events or summary measures	8–9	Overall stunting based on height for age was present in 192, i.e., 5% of the total cohort. Of these, 106 (55.2%) were male, and 86 (44.8%) were female. Most stunted children, 189 (98.4 %), were 4–10 years old, and 3(1.6%) were in the 10–14-year group. A total of 165(4.3%) Children were overweight; most of these 163(6.7%) were in the 11–14 years group. The number of underweight children was 8 (0.2%). Z score for height was -0.83 ± 0.77 (Mean ± S.D.) and -0.97 ± 0.91 in males and females, respectively, in the 4–10 years age group, and was 0.52 ± 0.74 and 0.44 ± 0.61 in males and females respectively in the 11–14 years group
Main results	16	(*a*) Give unadjusted estimates and, if applicable, confounder-adjusted estimates and their precision (eg, 95% confidence interval). Make clear which confounders were adjusted for and why they were included	7–10	To handle the issue of zero counts in some cells for categorical variables, we used a statistical method that accounted for this issue. Specifically, we used exact logistic regression, which calculates the exact probability of observing the observed data or data more extreme, given the null hypothesis of no association between the predictor and the outcome variable.

Continued on next page

		(*b*) Report category boundaries when continuous variables were categorized	7–8	Age<br>4–10<br>11–14<br>Frequency of Meal<br>≤ 2<br>>2<br>Hours of physical activity<br>Nil<br>≤ 1 hour<br>>1 hour<br>Height for Age<br>Stunted<br>Normal<br>Tall<br>Weight for Age<br>Underweight<br>Normal<br>Overweight
		(*c*) If relevant, consider translating estimates of relative risk into absolute risk for a meaningful time period	Not Applicable	Not Applicable
Other analyses	17	Report other analyses done—eg analyses of subgroups and interactions, and sensitivity analyses	Not Applicable	Not Applicable

Continued on next page

**Discussion**			**12–14**	The study found a high prevalence of unhealthy lifestyle habits, such as regular junk food intake and low physical activity, among school children. The prevalence of stunting was 5%, with a strong association with low socioeconomic status and junk food intake. Overweight cases were observed in 4.3% of children, with higher rates in the 11–14 age group. Urgent interventions are needed to address these issues.
Key results	18	Summarise key results with reference to study objectives	7–10	Most children, 2447 (63.8%), were 11–14 years old, and 1387 (36.2%) were 4–10. The mean height was 143.71± 16.51 centimetres, the mean weight was 36.5 ± 12.9 kilogram, and the mean BMI was 17.16±3.52. Multivariate logistic regression status and junk food combined affected stunting socioeconomic status was significantly associated with underweight p value=0.001.
Limitations	19	Discuss limitations of the study, taking into account sources of potential bias or imprecision. Discuss both direction and magnitude of any potential bias	16–17	The absence of randomization in school selection constrained the study's generalizability. However, the schools that gave their assent demonstrated a wide range in the student's socioeconomic status, representing Pakistani society. Children who do not have access to education were not taken into account in the study design. Undernutrition and obesity coexist among schoolchildren in Pakistan.
Interpretation	20	Give a cautious overall interpretation of results considering objectives, limitations, multiplicity of analyses, results from similar studies, and other relevant evidence		it is difficult to generalize the results due to the small number of participants residing at a single institution.
Generalisability	21	Discuss the generalisability (external validity) of the study results		

Continued on next page

**Other information**				
Funding	22	Give the source of funding and the role of the funders for the present study and, if applicable, for the original study on which the present article is based	Not Applicable	Not Applicable

Note: (1) *Give information separately for exposed and unexposed groups. (2) An Explanation and Elaboration article discusses each checklist item and gives methodological background and published examples of transparent reporting. The STROBE checklist is best used in conjunction with this article (freely available on the Web sites of PLoS Medicine at http://www.plosmedicine.org/, Annals of Internal Medicine at http://www.annals.org/, and Epidemiology at http://www.epidem.com/). Information on the STROBE Initiative is available at www.strobe-statement.org.

Supplementary III. List of Schools for Data collection.

1. Beaconhouse School, F-6/2 Islamabad.
2. Beaconhouse School, F-7/4 Islamabad.
3. Dar-e-Arqam School System, Rawalpindi.
4. FG Model School, Westridge.
5. Sir-Syed School Rawalpindi.
6. Govt. High School Gujar Khan.
7. City School Gujranwala.
8. New Mellinium Science Campus, More Eminabad.
9. Greens High School, Kalar Kahar.
10. Dar-e-Arqam, Kunjah, Gujrat.
11. Govt High School Greebpura, Gujrat.
12. Narowal English High School.
13. Allied School Satellite Town, Rawalpindi
14. Frobels National, Islamabad.
15. King Edward High School, Lahore.
16. St. Paul English High School, Lahore.
17. Govt. Community High School Mozang.
18. Govt. Islamia High School, Lahore.
19. City District Model Boys School, Kasur.
20. J.M Model Academy, Rawalpindi.
21. Quaid Model School, Islamabad.
22. Zawiya Academy, Rawalpindi.

AIMS Press

© 2023 the Author(s), licensee AIMS Press. This is an open access article distributed under the terms of the Creative Commons Attribution License (http://creativecommons.org/licenses/by/4.0)
